# Assessment of Serum Cortisol Levels in Hypothyroidism Patients: A Cross-Sectional Study

**DOI:** 10.7759/cureus.50199

**Published:** 2023-12-08

**Authors:** Seema R Sinha, Prem Prakash, J. R. Keshari, Rekha Kumari, Ved Prakash

**Affiliations:** 1 Department of Biochemistry, Indira Gandhi Institute of Medical Sciences, Patna, IND; 2 Department of General Surgery, Indira Gandhi Institute of Medical Sciences, Patna, IND; 3 Department of Endocrinology, Indira Gandhi Institute of Medical Sciences, Patna, IND

**Keywords:** adrenal glands, hpa axis, tsh levels, serum cortisol, overt hypothyroidism

## Abstract

Background: Hypothyroidism, characterized by insufficient thyroid hormone production, affects a significant global population, particularly women and the elderly. Recent research has emphasized the interaction between hypothyroidism and the hypothalamic-pituitary-adrenal (HPA) axis, highlighting cortisol's crucial role in the disease's physiological manifestations.

Objective: This study aims to evaluate serum cortisol levels in hypothyroid patients, examining the intricate relationship between these two endocrine systems. By exploring the potential impact of altered cortisol levels on hypothyroidism's clinical presentation and progression, the study seeks to contribute valuable insights to enhance diagnostic approaches and develop more effective treatment strategies.

Methods: A cross-sectional observational study was conducted at the Indira Gandhi Institute of Medical Sciences, assessing 65 hypothyroid cases and 65 age-matched euthyroid controls. Demographic data, medical history, and blood samples were collected, and serum cortisol, thyroid-stimulating hormone (TSH), triiodothyronine (T3), and thyroxine (T4) levels were measured. The study adhered to ethical considerations and received institutional approval.

Results: The study included 65 hypothyroid cases (56 females, 9 males) and 65 euthyroid controls. Serum cortisol showed a significant correlation with TSH and T4 levels. Linear regression revealed a negative correlation between serum T4 and T3 levels and serum cortisol in hypothyroidism. A positive correlation was observed between TSH and cortisol. These findings align with previous studies, suggesting potential regulatory mechanisms and compensatory responses in hypothyroid patients.

Discussion: The study's results emphasize the complex interaction between cortisol and thyroid function, suggesting a direct relationship between serum cortisol and TSH levels in hypothyroidism. Patients with severe hypothyroidism exhibited elevated cortisol concentrations, indicating a potential compensatory mechanism initiated by the HPA axis. Integrating serum cortisol assessment with conventional thyroid function tests could offer comprehensive insights into hypothyroidism severity and progression, providing a more holistic approach to patient care.

Conclusions: This study contributes to understanding the complex relationship between serum cortisol levels and hypothyroidism, emphasizing the need for further research to uncover underlying mechanisms and therapeutic implications. A comprehensive understanding holds the potential for more tailored and effective treatment strategies for individuals with hypothyroidism.

## Introduction

A significant percentage of people worldwide suffer from hypothyroidism, a common endocrine condition marked by insufficient production of thyroid hormones. The condition is more common in women and the elderly. Although there has been much research on the effects of hypothyroidism on the thyroid gland and the ensuing metabolic disorders, more recent studies have concentrated on the interaction between thyroid function and the hypothalamic-pituitary-adrenal (HPA) axis. The convoluted association between hypothyroidism and systemic physiological alterations has revealed the cortisol hormone to be a critical component. The hormone is a vital mediator of the body's stress response and regulates numerous metabolic processes.

Thyroid hormones, namely triiodothyronine (T3) and thyroxine (T4) are critical for both basal metabolism and the operation of nearly all bodily tissues and systems [1]. A decrease in thyroid hormone synthesis and thyroid gland function, which results in a slowed metabolism, is known as hypothyroidism [2]. Blood thyroid stimulating hormone (TSH) and T4 levels are assessed during a thyroid function test [3,4]. In women, overt hypothyroidism is estimated to be 1-2% prevalent, while in men, it is 0.1% [5].

Recent attention has been given to subclinical hypothyroidism, defined as a TSH elevation with T4 and T3 levels still within the normal range. Subclinical hypothyroidism is a common disorder; two large population-based studies revealed that 4% to 8.5% of individuals without known thyroid disease have subclinical hypothyroidism as evidenced by a mildly elevated TSH (i.e., 4.5-10 uIU/L) [6]. Stress and low blood glucose levels cause the steroid hormone cortisol to be released. Additionally, it was noted that whereas cortisol was high in the presence of primary hypothyroidism (elevated TSH), TSH was decreased [7] in the presence of primarily elevated cortisol.

Elevated cortisol levels are caused by hypothyroidism due to reduced cortisol clearance and negative cortisol feedback on the HPA. T3 and T4 secretion is kept within relatively narrow limits via a sensitive negative feedback loop in which TSH increases thyroid hormone synthesis and release. Cortisol promotes the synthesis and release of adrenocorticotropic hormone via a negative feedback process. Hypothyroidism is more common in elderly people than in younger people, especially in women, owing to the increasing incidence and prevalence of autoimmune thyroiditis [8].

The complex interrelation between the thyroid and adrenal systems has long fascinated researchers, with growing evidence suggesting a potential bidirectional influence. Moreover, the dysregulation of cortisol levels in hypothyroid patients has garnered significant attention, as altered cortisol dynamics might contribute to the clinical manifestations and complications associated with hypothyroidism. Understanding the subtle interaction between serum cortisol and hypothyroidism not only holds implications for comprehending the pathophysiology of the disease but also presents opportunities for refining diagnostic approaches and developing more effective treatment strategies.

The aim of this study is to evaluate the serum cortisol levels in patients diagnosed with hypothyroidism, shedding light on the complex relationship between these two endocrine systems. By examining the potential impact of altered cortisol levels on the clinical presentation and progression of hypothyroidism, this study aims to contribute to the growing body of knowledge concerning the complex hormonal interplay and its implications for patient management and care. The findings of this evaluation hold the promise of providing valuable insights that could pave the way for improved therapeutic interventions and personalized treatment modalities for individuals living with hypothyroidism.

## Materials and methods

This is a cross-sectional observational study designed to assess the serum cortisol levels in patients diagnosed with hypothyroidism. The study was conducted at the Department of Biochemistry, Department of Endocrinology, and Department of General Surgery Indira Gandhi Institute of Medical Sciences (IGIMS), Patna, India over a period of five months from July 2023 to November 2023. The study included 65 cases and 65 apparently healthy euthyroid subjects as control over the age of 18 years to 70 years who met the inclusion and exclusion criteria and visited the Department of Endocrinology and Department of General Surgery as outpatients and inpatients during the five-month period. Cases were identified by applying the American Thyroid Association (ATA) criteria for diagnosis of hypothyroidism. Demographic information, including age, gender, and relevant medical history, was collected for each participant. Age between 18 years and 70 years, with a confirmed diagnosis of primary hypothyroidism, and age and gender-matched healthy euthyroid subjects were included as controls in this study. Exclusion criteria were patients on thyroxin treatment, a history of smoking in the last year, subjects with heart disease, diabetes, stroke, or other neurological disorders or depression, significant medication use (beta-blockers, inhaled beta-agonists, hormonal contraceptives, corticosteroids) within prior three months, psychotropic medication use within prior eight weeks psychiatric hospitalization within the last one year patient having HIV.

Ethical considerations

Ethical approval for this study was obtained from the Institutional Ethics Committee, Indira Gandhi Institute of Medical Sciences (IGIMS), Patna (1112/IEC/IGIMS/2023). Informed consent was obtained from each participant before their inclusion in the study. Confidentiality and data protection protocols were strictly adhered to throughout the research process.

Blood sample collection and processing

Blood samples were collected from each participant in the morning hours to minimize diurnal variations in cortisol levels after a noted 8 hours to 10 hours of fasting, the venous blood sample was drawn to measure thyroid profile and serum cortisol. The blood sample collected was allowed to clot by placing it in a rack at room temperature for at least 30 minutes and a maximum of one hour. Then it was centrifuged at 3,000 rpm for five minutes, and the separated serum sample was stored at -20 degrees Celsius until used. Proper labeling and documentation of the samples were maintained to ensure accurate tracking and analysis.

Biochemical analysis

The clear serum obtained from the whole blood was analyzed for TSH, free T3, and free T4 on Abbott ARCHITECT i2000SR (Abbott, Chicago, USA) using the chemiluminescent microparticle immunoassay (CMIA) technology. Serum cortisol level was analyzed on Abbott ARCHITECT i1000SR (Abbott, Chicago, USA) using the CMIA technology. All the assays were performed following the manufacturer's instructions strictly.

Quality control

Quality control measures were implemented throughout the study, including regular calibration of laboratory equipment, adherence to standard operating procedures, and the inclusion of internal controls to ensure the accuracy and reliability of the results.

Statistical analysis

Statistical analysis was carried out using STATA software, Version 17 (StataCorp LLC, College Station, Texas, USA). Descriptive statistics were generated to enable comparisons between groups. Distributions were compared using Student’s t-test. Categorical variables were presented as numbers and percentages and were compared using the chi-square test. Pearson’s correlation was used to find out the relationship between different continuous variables. Multivariate logistic regression analysis was applied to evaluate the effect of thyroid function parameters on the level of serum cortisol. A p-value of <0.05 was considered statistically significant.

## Results

In a total of 65 cases, 9 were males and 56 were females. Age and sex-matched 65 healthy (euthyroid) controls were included in this study. Table 1 lists the biochemical and socio-demographic characteristics of the study participants along with the normal function test range as per ATA guidelines [9].

**Table 1:** Lists of the biochemical and socio-demographic characteristics of the study participants BMI: Body mass index; TSH: Thyroid stimulating hormone; T3: Triiodothyronine; T4: Thyroxine

	Normal range	Case (n = 65)	Control (n = 65)	p-value
Age (years)	NA	40.41 ± 15.25	37.72 ± 12.67	>0.05
BMI (kg/m^2^)	18.5-24.9 kg/m^2^	24.16 ± 3.002	24.25 ± 4.59	>0.05
TSH (uIU/L)	0.4-5.5 uIU/L	26.44 ± 9.42	4.62 ± 1.71	<0.05
T3 (ng/ml)	0.8-2 ng/mL	0.62 ± 0.10	1.51 ± 0.32	<0.05
T4 (μg/dl)	5.0-11.0 μg/dL	5.38 ± 1.21	8.22 ± 1.86	<0.05
Cortisol (μg/dl)	5-25 μg/dL	59.98 ± 10.14	14.01 ± 5.93	<0.05

**Figure 1:**
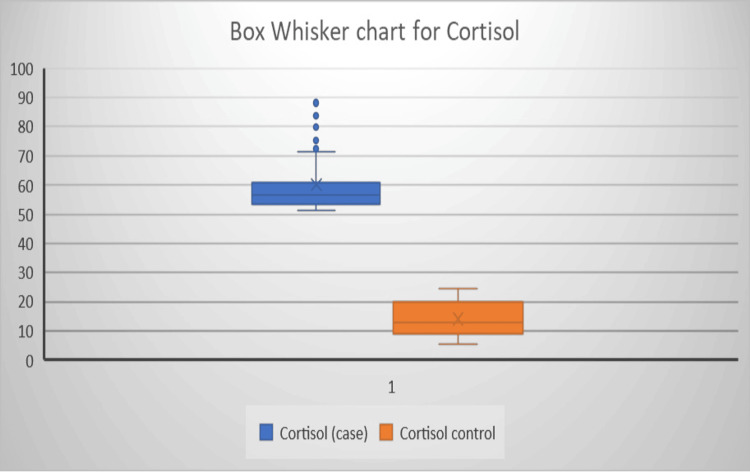
Box whisker chart showing a significant difference in serum cortisol levels between the case and control group (p < 0.05)

The mean age in the case group and control group were 40.41 ± 15.25 and 37.72 ± 12.67 years. There was no significant difference between the case and control group for age and sex. Linear regression was carried out to analyze the independent factors associated with the most significant change in TSH, T3, T4, and cortisol levels. The mean serum cortisol level in the case group was 59.98 ± 10.1 μg/dl. Figure 1 illustrates the Box whisker chart showing a significant difference in serum cortisol levels between the case and control group (p < 0.05).

There was a strong positive correlation between the serum cortisol and TSH (r = 0.9571), and a strong negative correlation between the serum cortisol, T3, and T4 having r = -0.9491 and -0.9751, respectively, as shown in Table 2.

**Table 2:** The correlation of serum cortisol levels with T3, T4, and TSH TSH: Thyroid stimulating hormone; T3: Triiodothyronine; T4: Thyroxine

Serum cortisol (μg/dl)	Correlation
T3 (ng/ml)	-0.9491
T4 (μg/dl)	-0.9751
TSH (uIU/L)	0.9571

**Figure 2:**
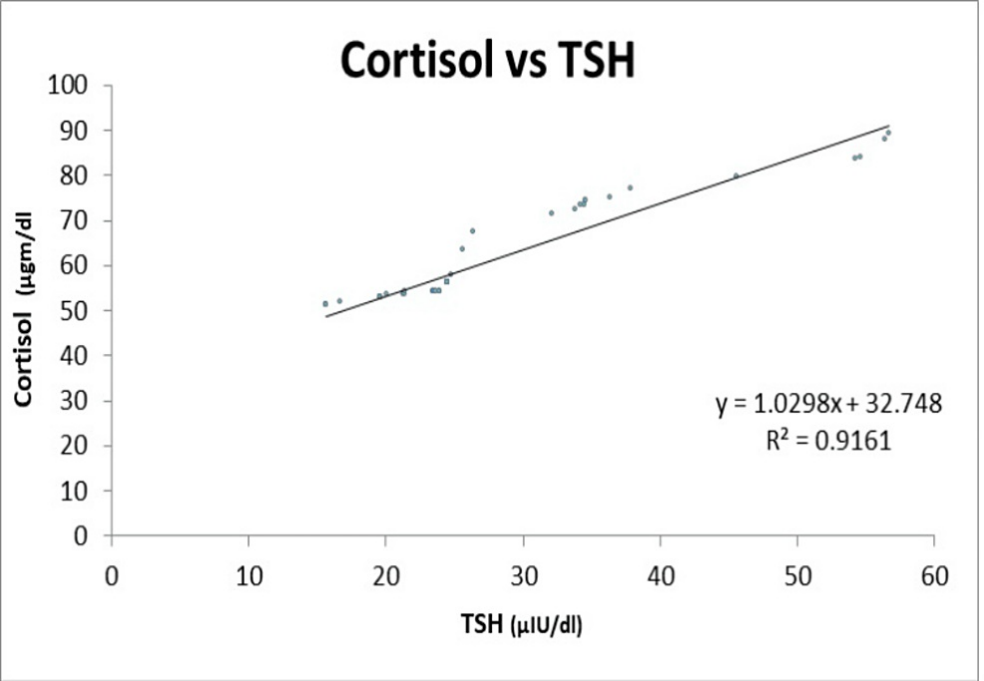
Positive correlation between TSH and cortisol TSH: Thyroid stimulating hormone

Figure 2 illustrates a strong positive correlation between TSH and cortisol. Results are presented as the correlation coefficient.

**Figure 3:**
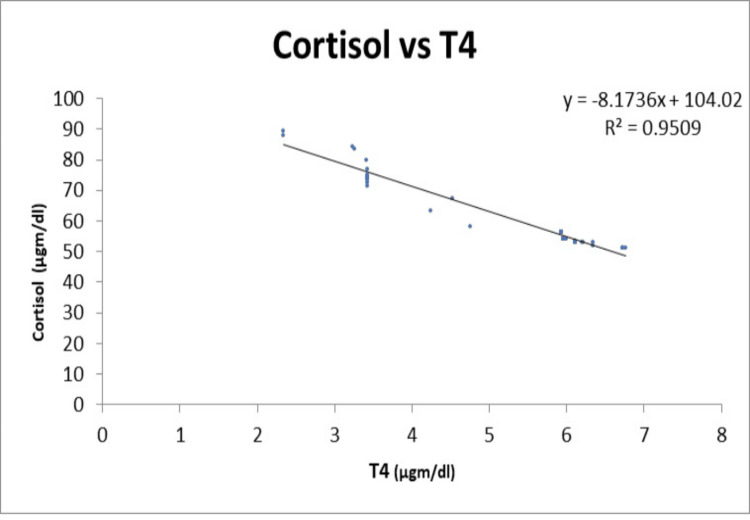
Illustration of a negative linear correlation between serum T4 and serum cortisol levels in the hypothyroidism group T4: Thyroxine

**Figure 4:**
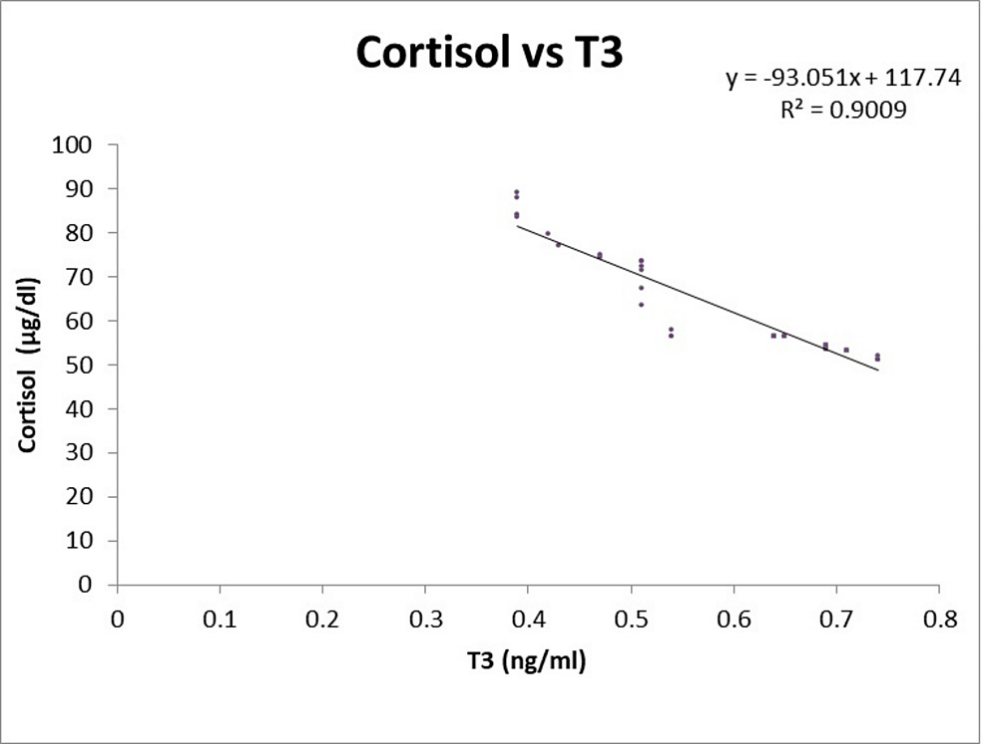
Illustration of a negative linear correlation was found between serum T3 and serum cortisol levels in the hypothyroidism group T3: Triiodothyronine

Figures 3, 4 illustrate a negative linear correlation between serum T4 and serum T3 and serum cortisol levels in the hypothyroidism group.

## Discussion

The present study aimed to evaluate the serum cortisol levels in patients diagnosed with hypothyroidism, shedding light on the convoluted relationship between these two endocrine systems. Our findings revealed several key insights into the interplay between cortisol dynamics and thyroid function, underscoring the significance of considering the HPA axis in the context of hypothyroidism.

Our results demonstrated a statistically significant correlation between serum cortisol levels and markers of thyroid dysfunction, particularly TSH and T4 levels. Notably, we observed a direct relationship between serum cortisol and TSH, suggesting a potential regulatory mechanism between the HPA axis and thyroid function. These findings are in accordance with previously done similar studies [2-4]. This finding aligns with previous research highlighting the complex feedback mechanisms between the hypothalamus, pituitary gland, adrenal cortex, and thyroid gland, emphasizing the intricate crosstalk between these endocrine systems. Furthermore, our study indicated that patients with more severe hypothyroidism, as evidenced by higher TSH levels and lower T4 levels, exhibited elevated serum cortisol concentrations. The findings are similar to a study by Iranmanesh et al. [5]. They also found a significant increase in serum cortisol levels in primary hypothyroidism. Reduced metabolic clearance of cortisol and a presumed reduction in the negative feedback impact of cortisol on the HPA may be the main causes of this shift in serum cortisol [6]. This observation suggests a potential compensatory mechanism initiated by the HPA axis in response to the metabolic derangements associated with hypothyroidism. The upregulation of cortisol secretion in these patients might signify an adaptive response to mitigate the metabolic consequences of thyroid hormone deficiency.

Cortisol levels were elevated in hypothyroid individuals in the current investigation, and there was a positive association between TSH and cortisol, which is consistent with the findings of a previous similar study by Ali and Dhelal [10,11]. The therapeutic implications of our findings highlight the necessity of assessing the entire endocrine profile, including thyroid function and cortisol dynamics, in the therapy of hypothyroid patients. Our findings imply that measuring blood cortisol levels in addition to conventional thyroid function tests could provide useful insights into the severity and course of hypothyroidism, allowing for a more comprehensive approach to patient care and therapy.

## Conclusions

In conclusion, our findings underscore the complex relationship between serum cortisol levels and hypothyroidism, highlighting the intricate interplay between these two endocrine systems. Further research is warranted to elucidate the underlying mechanisms driving the observed associations and explore the therapeutic implications of modulating cortisol dynamics in hypothyroidism. A comprehensive understanding of the synergistic effects of these endocrine pathways holds the potential to inform more tailored and effective treatment strategies for individuals living with hypothyroidism.
